# Genetic Variability and Population Structure of Ethiopian Sesame (*Sesamum indicum* L.) Germplasm Assessed through Phenotypic Traits and Simple Sequence Repeats Markers

**DOI:** 10.3390/plants10061129

**Published:** 2021-06-02

**Authors:** Desawi Hdru Teklu, Hussein Shimelis, Abush Tesfaye, Jacob Mashilo, Xiurong Zhang, Yanxin Zhang, Komivi Dossa, Admire Isaac Tichafa Shayanowako

**Affiliations:** 1 African Centre for Crop Improvement, University of KwaZulu-Natal, Pietermaritzburg 3209, South Africa; Shimelish@ukzn.ac.za (H.S.); jacobmashilo.jm@gmail.com (J.M.); shayanowako@gmail.com (A.I.T.S.); 2 Ethiopian Agricultural Transformation Agency, Addis Ababa 708, Ethiopia; 3 International Institute of Tropical Agriculture, Ibadan 5320, Nigeria; At.Abebe@cgiar.org; 4 Oil Crops Research Institute, Chinese Academy of Agricultural Sciences, Key Laboratory of Biology and Genetic Improvement of Oil Crops, Ministry of Agriculture, Wuhan 430062, China; zhangxr@oilcrops.cn (X.Z.); zhangyanxin@caas.cn (Y.Z.); komiri.dossa@ucad.edu.sn (K.D.); 5 Laboratory of Genetics, Horticulture and Seed Sciences, Faculty of Agronomic Sciences, University of Abomey-Calavi, Cotonou 01 BP 526, Benin

**Keywords:** agronomic traits, Ethiopia, genetic diversity, microsatellites, population structure, principal component analysis, *Sesamum indicum*

## Abstract

Ethiopia is one of the centers of genetic diversity of sesame (*Sesamum indicum* L.). The sesame genetic resources present in the country should be explored for local, regional, and international genetic improvement programs to design high-performing and market-preferred varieties. This study’s objective was to determine the extent of genetic variation among 100 diverse cultivated sesame germplasm collections of Ethiopia using phenotypic traits and simple sequence repeat (SSR) markers to select distinct and complementary genotypes for breeding. One hundred sesame entries were field evaluated at two locations in Ethiopia for agro-morphological traits and seed oil content using a 10 × 10 lattice design with two replications. Test genotypes were profiled using 27 polymorphic SSR markers at the Oil Crops Research Institute of the Chinese Academy of Agricultural Sciences. Analysis of variance revealed significant (*p* ≤ 0.05) entry by environment interaction for plant height, internode length, number of secondary branches, and grain yield. Genotypes such as Hirhir Kebabo Hairless-9, Setit-3, Orofalc ACC-2, Hirhir Humera Sel-6, ABX = 2-01-2, and Setit-1 recorded grain yield of >0.73 ton ha^−1^ with excellent performance in yield component such as oil yield per hectare. Grain yield had positive and significant (*p* < 0.01) associations with oil yield (*r* = 0.99), useful for simultaneous selection for yield improvement in sesame. The SSR markers revealed gene diversity and polymorphic information content values of 0.30 and 0.25, respectively, showing that the tested sesame accessions were genetically diverse. Cluster analysis resolved the accessions into two groups, while population structure analysis revealed four major heterotic groups, thus enabling selection and subsequent crossing to develop breeding populations for cultivar development. Based on phenotypic and genomic divergence, the following superior and complementary genotypes: Hirhir Humera Sel-6, Setit-3, Hirhir Kebabo Hairless Sel-4, Hirhir Nigara 1st Sel-1, Humera-1 and Hirhir Kebabo Early Sel-1 (from cluster II-a), Hirhir kebabo hairless-9, NN-0029(2), NN0068-2 and Bawnji Fiyel Kolet, (from cluster II-b). The selected genotypes will serve as parents in the local breeding program in Ethiopia.

## 1. Introduction

Sesame (*Sesamum indicum* L.) is a multi-purpose high-value oilseed crop. It is a global commodity serving the food, feed, and cosmetic industries. The seed oil content of sesame is about 60%, the highest when compared with other oilseed crops such as sunflower (~45%), rapeseed (~40%), and soybean (~20%) [1,2,3,4]. Sesame oil comprises about 85% unsaturated and 15% saturated fatty acids. The fatty acid contains linoleic acid (~46%), oleic acid (~38%), palmitic acid (~12%), and stearic acid (~4%) [4,5,6,7]. Sesame seed is a rich source of protein (≈24%), carbohydrate (≈13.5%), vitamins (e.g., A and E), lignans (sesamin and sesamolin), γ tocopherol, phytosterols (β-sitosterol and Campesterol), policosanols (Docosanol, Tetracosanol, Hexacosanol, and Octacosanol) and lipids [4,7,8,9]. These attributes make sesame a ‘superfood’ comprising all the essential human nutrients in desirable proportions.

Sesame is the second most valuable export crop after coffee (*Coffea arabica* L.) and a major contributor to Ethiopia’s gross domestic product [10]. In Ethiopia, the area allocated for sesame production in 2018 was 294,819.49 ha, approximately 39.4% of the total estimated area allocated for oil crops production [11]. Compared with global sesame production, Ethiopia ranks eight with a total annual output of 301,302 tons after Sudan (981,000 tons), Myanmar (768,858 tons), India (746,000 tons), Nigeria (572,761 tons), Tanzania (561,103 tons), China (433,386 tons), and China Mainland (431,500 tons) [12].

Ethiopia is the center of origin and diversity for the cultivated sesame and its allied species. The Ethiopian Biodiversity Institute (EBI) maintains one of the most extensive core collections of sesame genetic resources in Africa. About 5000 genetically diverse sesame germplasm resources are conserved by the EBI [13]. The germplasm pool can provide various unique economic traits and gene combinations for global sesame improvement. However, the genetic resources maintained at the EBI are yet to be explored for local, regional, and international sesame improvement programs to develop high-performing and market-preferred varieties. Ethiopia’s mean sesame yield is 0.68 tons ha^−1^, which is relatively low compared with a mean yield of 1 ton ha^−1^ in sub-Saharan Africa and 1.29 ton ha^−1^ in Egypt [11,12]. The low productivity is attributable to a lack of improved and high-yielding varieties and traditional production technologies, among other constraints. Landrace varieties are the main sources of seed for cultivating the crop in Ethiopia. Landraces are inherently low yielders and prone to capsule shattering leading to reduced productivity. However, landraces are highly valued for possessing intrinsic farmer-preferred attributes such as unique taste and aroma, adaptation to marginal growing conditions that often characterize low input farming systems [8,14].

Sesame genetic resources maintained at the EBI can be explored to search for new sources of useful genetic variation for economic traits. This includes grain yield and yield- components, resistance to diseases and insect pests, tolerance to abiotic stresses, capsule shattering tolerance, and nutritional quality. This will identify desirable and complementary parents and for gene discovery. Hence, rigorous phenotyping and genotyping can establish genetic polymorphism in the germplasm pool and classify the heterotic groups for ideotype breeding.

Previous studies have reported considerable phenotypic variation for agronomic and quality traits in Ethiopia’s sesame genetic resources [15,16,17,18]. However, these studies did not fully represent the landrace collections from various parts of Ethiopia. Hence there is a need for a comprehensive assessment of the genetic diversity present in the Ethiopian sesame using a relatively more significant number of accessions representing the diverse germplasm resources and sampled from various regions through phenotypic traits and effective molecular markers.

Several molecular markers such as amplified fragment length polymorphism (AFLP), restriction fragment length polymorphism (RFLP), random amplified polymorphic DNA (RAPD), microsatellites or simple sequence repeat (SSR), and single nucleotide polymorphisms (SNPs) markers are widely used in genetic diversity analysis of various crop genetic resources. SSR or microsatellites have been commonly used in genetic variation studies on sesame [19,20,21,22]. The SSRs are preferred for their ability to detect higher degrees of polymorphism, higher reproducibility, and abundant coverage of the genome [20,21]. Moreover, SSR markers can be used for loci with multiple co-dominant alleles [23]. Wei et al. [20] and Asekova et al. [21] assessed genetic diversity and population structure present in sesame genetic resources sampled from China and Korea using 44 and 23 SSRs, respectively. The authors reported two and three major heterotic groups among the Chinese and Korean collections, respectively. The level of genetic diversity varies among different germplasm populations and environmental conditions, suggesting that each set of populations must be assessed in a target production environment for selection and genetic grouping. Therefore, this study’s objectives were to determine the extent of genetic variation among 100 diverse sesame germplasm collections of Ethiopia using phenotypic traits and simple sequence repeat markers to select and recommend distinct and complementary parents for direct production, breeding, and conservation.

## 2. Materials and Methods

### 2.1. Plant Materials

The study used a mini-core collection of 100 sesame entries originally collected from the Amhara, Tigray, Afar, Oromia, and Gambela regions in Ethiopia. The test genotypes were obtained from the sesame and groundnut breeding program of Werer Agricultural Research Centre of the Ethiopian Institute of Agricultural Research (EIAR). The collection comprised of 95 accessions, one landrace (farmer variety), and four released varieties. The landrace variety ‘‘Hirhir” is widely cultivated by farmers in the study areas. The four released varieties (i.e., Setit-1, -2, -3, and Humera-1) were developed by the Humera Agricultural Research Center (HuARC) through mass selection amongst the local germplasm collections. The details of the germplasm collections used in the study are summarized in Table 1.

### 2.2. Phenotyping

#### 2.2.1. Description of the Study Sites

The study was conducted in northwestern Ethiopia in two selected locations, namely, Humera (14°15′ N, 36°37′ E) and Kebabo (13°36′ N, 36°41′ E). Humera and Kebabo are agricultural research stations of the Humera Agricultural Research Centre of EIAR and the Tigray Agricultural Research Institute (TARI). The two sites represent the major sesame production environments in Ethiopia. Humera and Kebabo are situated at an altitude of 609 and 696 m above sea level and receive a total rainfall of 576.4 and 888.4 mm, respectively. The mean minimum and maximum temperatures at Humera site range from 20.3 to 36.5 °C. Kebabo has an average minimum and maximum temperatures of 16.9 and 31.7 °C. The two sites have predominantly clay soil [24].

#### 2.2.2. Experimental Design and Trial Management

The experiment was conducted under field conditions and laid out using a 10 × 10 simple lattice design, with two replications, at each site. Each entry was planted in four rows plots measuring four meters in length, with an inter-row and intra-row spacings of 0.4 m and 0.1 m, respectively. The trials were maintained following the standard agronomic practices of sesame production [24].

#### 2.2.3. Phenotypic Data Collection

Data were collected on quantitative and qualitative traits. Plant height, internode length, number of primary branches per plant, number of secondary branches per plant, number of capsules per plant, number of seeds per capsule, stem height to first branch, and distance from lowest branch to 1st capsule were recorded from 10 randomly selected and tagged plants during plant growth and at harvest. Plant height (PH) was measured from the base to the tip of the plant. Stem height from the base to the 1st branch (SHB) was measured from the base of the plant to first emerged primary branch. Internode length (INL) was measured between two consecutive nodes situated in the middle of the plant. The number of primary branches per plant (NPB) was counted from the plant’s main stem, while the number of secondary branches per plant (NSB) was counted from the plant’s main branch. Distance from the base of the lowest branch to the first capsule (DFLBC) was measured as the distance between the lowest situated primary branch to the 1st emerged capsule on the main stem and expressed in cm.

The number of days to flowering (DF) was recorded by counting the number of days from planting to the date when 50% of the plants showed flowers, while days to maturing (DM) was recorded as the number of days from planting to the date when 75% of the plants reached physiological maturity. The number of capsules per plant (NCPP) and number of seeds per capsule (NSPP) were counted from a composite of three capsules per plant at harvest. Thousand seed weight (TSW) was measured from a random sample of 1000 seeds of each entry. Grain yield (GYH) was measured in grams per plot and converted into ton (t) per hectare (ha^−1^).

Oil content was determined at Wuhan city, China using the Near-Infrared Spectroscopy (NIR) (FOSS, model DS2500, Hillerød, Denmark). Oil yield per hectare was calculated and expressed in tons per hectare as the product of grain yield and percent oil content.

#### 2.2.4. Phenotypic Data Analysis

The phenotypic data were subjected to analysis of variance (ANOVA) using the alpha-lattice and general linear model (GLM) procedures of the SAS software version 9.4 [25]. A combined analysis of variance across the two locations was performed after Bartlett’s homogeneity test of variance. Mean comparisons among accessions were performed using Tukey’s Honestly Significant Difference (HSD) test procedure at 5% level of significance used to identify significant differences among means in Table 4. The correlation among traits was performed using R software version 4.0 [26] to determine the magnitude of associations among the studied traits. Multivariate analysis using the principal components was performed using R software version 4.0 [26].

### 2.3. Genotyping

#### 2.3.1. DNA Extraction, Primer Selection, Polymerase Chain Reaction, and Electrophoresis

The above 100 sesame entries (Table 1) were planted at the Oil Crops Research Institute (OCRI)—the Chinese Academy of Agricultural Sciences (OCRI-CAAS), China. Ten seeds per entry were sown in a plastic tray in a growth room. Three two-weeks old seedlings were randomly selected from each entry, and fresh young leaves were collected and ground in liquid nitrogen for DNA extraction. The DNA was extracted following the Cetyl-tetramethyl ammonium bromide (CTAB) method. Approximately 200 mg of ground plant tissue combined with 500 µL of CTAB buffer was incubated in a water bath at 65 °C, 4 times for 10 min, and subjected to centrifugation at 12,000 rpm for 10 min at 4 °C. The supernatant was then transferred into new 5 mL micro-tubes, and 400 µL chloroform: iso-amyl alcohol (24:1) was added into the tubes and mixed gently. After a minute of centrifugation (centrifuged at 12,000 rpm for 10 min at 4 °C), the supernatant was transferred into new 5 mL micro-tubes, and 400 µL isopropanol was added into the tubes, mixed gently and kept at −20 °C for 30 min and subjected to centrifugation at 12,000 rpm for 10 min at 4 °C. The precipitated DNA was washed by 75% ethanol three times. The resulting pellet was dried under vacuum and dissolved in 100 uL DD H_2_O. DNA concentrations were measured using the Quantus TM Fluorometer (Promega Corporation, Madison, USA). Microsatellites from 13 linkage groups were designed and used for the following experiments. The 27 primers were selected because of their suitability in discriminating sesame genotypes. The presently used primers were initially selected amongst 160 candidate primers based on their higher polymorphic information content and provided clear and informative amplicon profiles in sesame genetic analysis [27].

The polymerase chain reaction (PCR) conditions were maintained as follows; each PCR reaction was carried out in a 20 μL solution containing 25 ng of DNA, 4 μmol of forward primers, 4 μmol of reverse primers, 1 × buffer, 0.25 mmol of dNTPs, and 0.80 U Taq polymerase. The temperature profile used for PCR amplification comprised a denaturation step at 94 °C for 1 min, followed by primer annealing temperature at 45.2–53 °C for 1 min, and elongation at 72 °C for 1 min. After 34 cycles, the reaction was terminated with a 10 min final extension time at 72 °C.

The PCR reaction conditions were the same for all the primers, except for the annealing temperatures. The PCR products were electrophoresed on 6% Acrylamide gel (=200 mL of 5 × T.B.E., 420 g of Urea [H2NCONH2], 75 g of Acrylamide, 3 g of Bis-Acrylamide, and 400 mL of distilled water) at a voltage value of 2000, current 300 A, power 80 W for 1:30 h. After silver staining, the bands on the gels were recorded, and a total of 27 markers (Table 2) with high polymorphism were used for capillary electrophoresis. The PCR products were separated by capillary electrophoresis on an ABI 3730 automatic sequencer. The marker data was presented as fragment sizes in an excel spreadsheet.

#### 2.3.2. Genotypic Data Analysis

The fragment sizes were determined using the ABI 3730 automatic sequencer. Data were analysed using the software GeneMarker V 2.2.0 to determine peak detection threshold levels that ranged from the minimum intensity of 500 and max intensity of 30,000. The 27 primers were used to detect the band sizes based on the peak detection thresholds, which were then scored using 1 to denote presence and 0 for absence. Genetic parameters, such as major allele frequency (M.A.F.), observed heterozygosity (Ho), expected heterozygosity (He), and the polymorphic information content (PIC) were calculated using Power Marker v3.2. Cluster analysis was carried out using a neighbor-joining (NJ) algorithm using the unweighted pair group method (UWPGM) in R software version 4.0 [26].

The population structure of the 100 sesame accessions was investigated using the Bayesian clustering method in STRUCTURE version 2.3.4 [28]. The length of the burn-in period and Markov Chain Monte Carlo (MCMC) were set at 20,000 iterations [29]. To obtain an accurate estimation of the number of populations, ten runs were performed for each K-value (assumed number of subpopulations), ranging from 1 to 10. Further, Delta K values were calculated, and the appropriate K value was estimated by implementing the [29] method using CLUMPK. The principal coordinate analysis was also used to deduce the genotypes’ genetic structure using Darwin version 6.

## 3. Results

### 3.1. Genetic Variation and Mean Performance of Sesame Accessions

Combined ANOVA revealed significant (*p* ≤ 0.05) entry x environment interaction for plant height, internode length, number of primary branches, number of secondary branches, distance from the base of the lowest branch to 1st capsule, and grain yield per hectare (Table 3). Entries showed significant (*p* ≤ 0.05) differences for days-to-50% flowering, days-to-75% maturity, plant height, internode length, number of secondary branches, number of seeds per capsule, distance from the base of the lowest branch to 1st capsule, and grain yield per hectare.

Based on grain yield response, the top 10 best performing and the five bottom performing accessions are summarized in Table 4. The mean grain yield across locations was 0.48 ton ha^−1,^ and the mean thousand-seed weight was 2.9 g. The highest grain yield was recorded for entries such as: Hirhir Kebabo Hairless-9 (1.01 ton ha^−1^), Setit-3 (0.84 ton ha^−1^), Orofalc ACC-2 (0.80 ton ha^−1^), Hirhir Humera Sel-6 (0.78 ton ha^−1^), ABX = 2-01-2 (0.74 ton ha^−1^), and Setit-1 (0.73 ton ha^−1^). These genotypes expressed high oil yields of 0.40, 0.40, 0.40, 0.39, 0.36, and 0.39 ton ha^−1^ than other test genotypes. The accessions Bawnji Fiyel Kolet, NN0056, Hirhir Humera Sel-8, NN-0068-1, and ACC-NS-010 had the highest oil content of 55.6, 55.2, 54.7, 54.6, and 54.10% than other genotypes, respectively. The five bottom performing accessions in terms of grain yield were NN-0183-3 (0.17 ton ha^−1^), NN-0020 (0.24 ton ha^−1^), NN-0108-2 (0.26 ton ha^−1^), NN00136-1 (0.26 ton ha^−1^), and NN-0143 (0.28 ton ha^−1^) with low oil yield of 0.07, 0.12, 0.04, 0.13, and 0.15 ton ha^−1^, in that order. These accessions yielded below-average grain and oil yields.

### 3.2. Correlations of Yield and Yield Components

Phenotypic correlation coefficients for the studied traits are presented in Table 5. Grain yield was significantly and positively correlated with oil yield (*r* = 0.99; *p* < 0.01). Significant and positive correlations were also observed between grain yield and internode length (*r* = 0.35; *p* < 0.01), number of secondary branches (*r* = 0.21; *p* < 0.01), number of capsules per plant (*r* = 0.18; *p* < 0.01), number of seeds per capsule (*r* = 0.17; *p* < 0.01), stem height from base to 1st branch (*r* = 0.16; *p* < 0.01), and thousand-seed weight (*r* = 0.23; *p* < 0.01).

### 3.3. Principal Component Analysis

**Figure 1:**
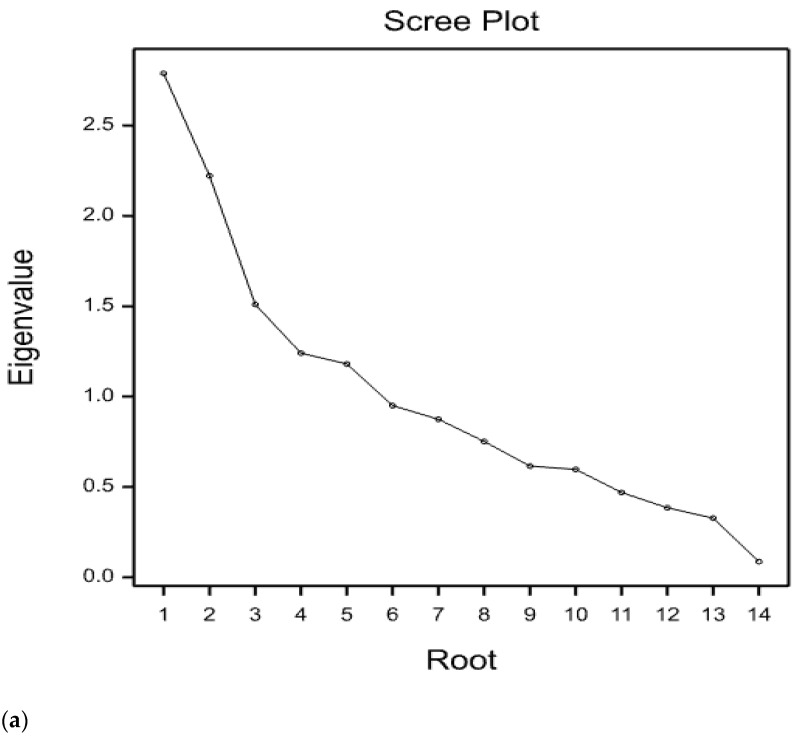

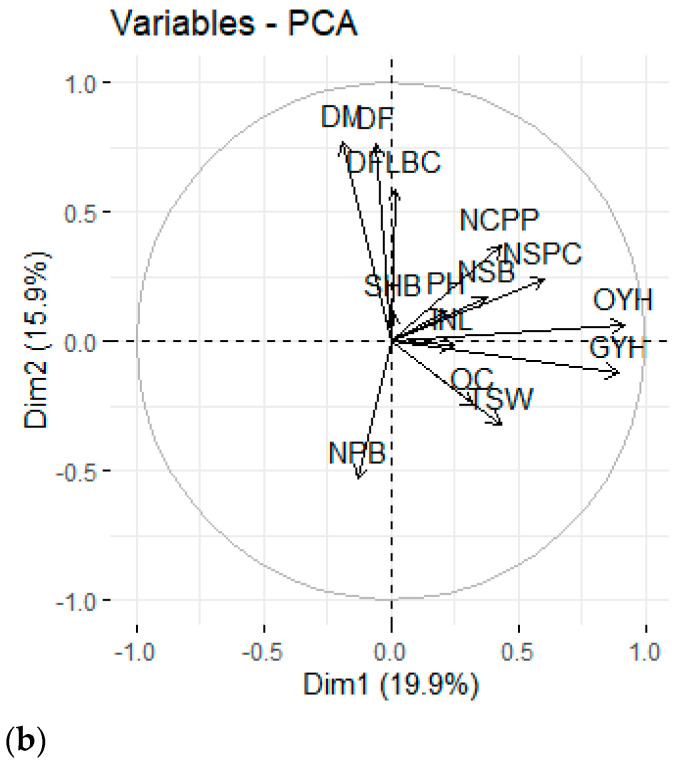
Principal component analysis based on agro-morphological traits, oil content and oil yield of 100 sesame germplasm collections. Note: Figure (**a**) is a scree plot indicating eigenvalues and percentage variation explained by principal components; while Figure (**b**) is a biplot that indicates sesame traits projection on the first two principal components. Days-to-50% flowering (DF), Days-to-75% maturity (DM), Plant height (PH) (cm), Inter node length (INL) (cm), Number of primary branches per plant (NPB), Number of secondary branches per plant (NSB), Number of capsules per plant (NCPP), Number of seeds per capsule (NSPC), Stem height to 1st branch (SHB) (cm), Distance from lowest branch to 1st capsule (DFLBC)(cm), Thousand-seed weight (TSW) (g/1000 seed), Oil content (OC) (%), Oil yield per hectare (OYH) (ton ha^−1^), Grain yield per hectare (GYH) (ton ha^−1^).

Principal component analysis (PCA) was computed to show each trait’s contribution to the overall observed variation. A scree plot was generated to visualize the number of principal components. Overall, four principal components were identified with >1 Eigen values of which principal components 1 (PC1) and PC2 explained the highest proportion to the total variance (Figure 1). Principal component one (PC1) explained 19.9% to the total variation with OYH and GYH contributing the largest variation to PC1. Principal component two (PC2) accounted for 15.9% of the total variation, and DM, DF, DFLBC, and NPB were the most influential traits.

### 3.4. Genetic Polymorphism of the SSR Markers

The summary statistics describing the SSR markers are presented in Table 6. The major alleles frequency per locus ranged from 0.52 to 0.96, with a mean of 0.78 alleles per locus. The observed heterozygosity varied from 0.08 to 0.96, with a mean of 0.43. The unbiased expected heterozygosity (gene diversity) of the markers ranged from 0.08 to 0.5, with a mean of 0.30. The PIC values ranged from 0.07 (for markers ID0041, ID0175, and ZMM2818) to 0.37 (ZMM3261 and ZMM1189) with a grand mean value of 0.25.

### 3.5. Population Structure Analysis

**Figure 2:**
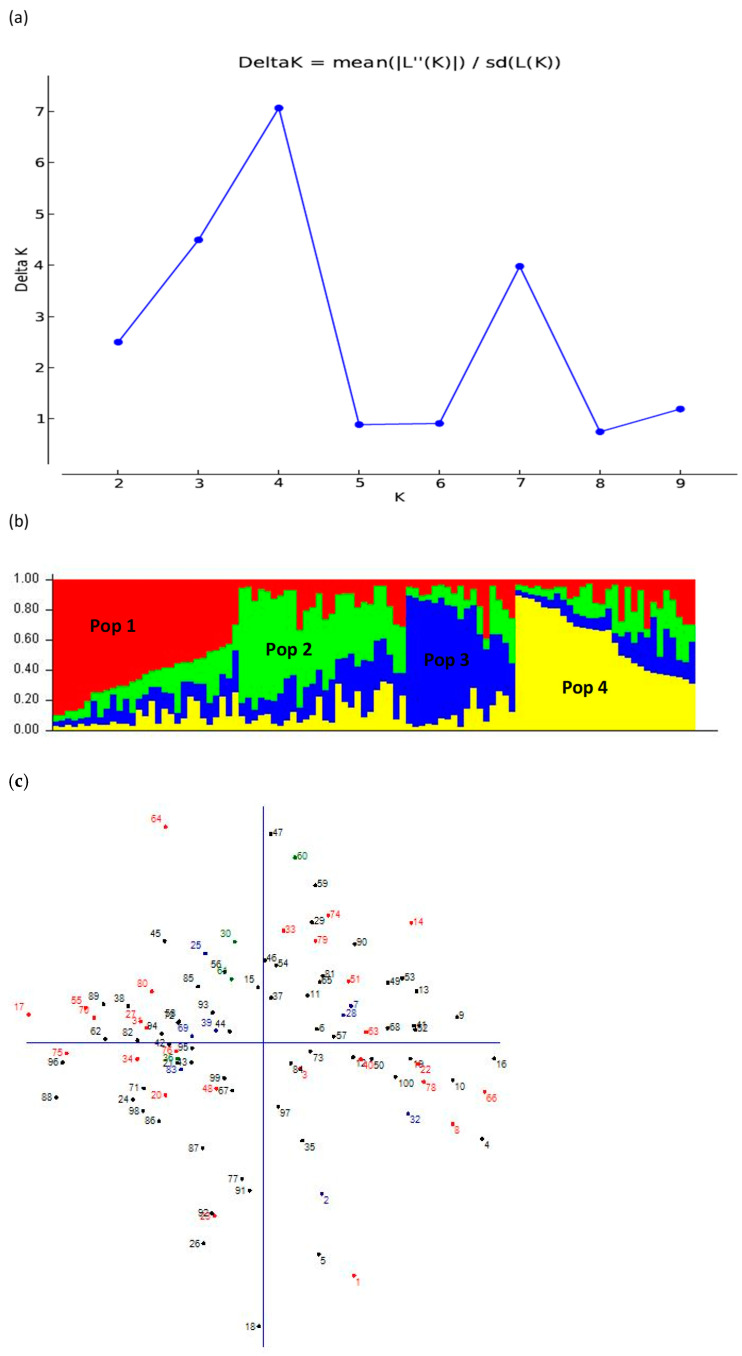
Subpopulation inference among100 sesame entries based on 27 SSR markers: (**a**) Delta K estimation based on the Evanno procedure, (**b**) Sub-populations for the best delta K value of four. Pop 1, 2, 3, and 4 denote Populations 1, 2, 3, and 4, respectively, and (**c**) principal coordinate clustering of genotypes.

Structure analysis revealed four populations amongst the 100 sesame entries (Figure 2b, Table 7). Sixty-three accessions were allocated to the four populations, whereas 37 accessions were admixtures with no specific membership (Table 7). Population I consisted of 24 accessions collected from the following regions: Amhara (17 collections), Tigray (3), Afar (3), and Oromia (1). Population II had 13 accessions initially collected from the Amhara region (8), Afar (4), and Tigray (1). Population III comprised nine accessions, sourced from the Amhara (5 accessions) and Tigray (4) regions. Population IV consisted of 17 accessions sourced from Tigray (10 accessions), Amhara (6), and Afar (1) regions. 

Entries allocated in population I had good branching ability (NN-0052, NN-0029-1, and GXT = 85(28-2)), many seeds per capsule (Gojam Azene (Aleka), and ABXT-85-Sel-2-1), and significant oil yield (NN-0026), and seed oil content (NN0064-1 and NN0071). 

Population II accessions were early maturing with tall plants. Some population II accessions had a significant number of seeds per capsule (NN0025 and ABX = 2-01-2), and grain yield(Orofalc ACC-2, and ABX = 2-01-2). In addition, population II comprises accessions such as NN0056, Hirhir Baeker-Sel-3, and Orofalc ACC-2 with good oil content. Genotypes allocated in population III were early maturing with taller plants. These accessions were outstanding in thousand-seed weight (e.g., Hirhir Filwha Large Seeded), grain yield (Hirhir Kebabo Hairless-9), oil yield (Hirhir Kebabo Hairless-9), and seed oil content ( Hirhir Kebabo Hairless-9).

Genotypes allocated in population IV were also early maturing with taller plants. Some accessions within this group also had remarkable seeds per capsule (Setit-3 and NN-0020), better thousand-seed weight (3.4 g), higher seed and oil yields (0.84 and 0.40 ton ha^−1^), and oil content (54.7%). To develop new breeding populations possessing desirable economic traits new crosses could be developed between the selected parents. Hence accessions Orofalc ACC-2 (from population II), Hirhir Filwha Large Seeded (population III), and Setit-3, Hirhir Humera Sel-6 (population IV) are ideal candidates with complementary traits for production and further breeding. However, the principal coordinate analysis assigned the 100 genotypes into admixture groups with an inconclusive structure (Figure 2c).

### 3.6. Cluster Analysis of 100 Sesame Accessions

**Figure 3:**
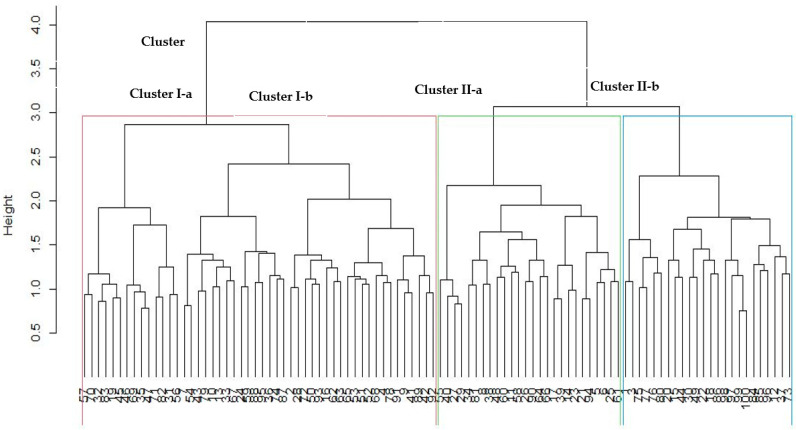
Dendrogram based on Unweighted Pair Group Method with Arithmetic Mean (UPGMA) showing the genetic relationship among 100 sesame entries using 27 SSR markers. Note: see Table 1 for codes of entries.

The cluster analysis involving 100 sesame genotypes resolved two clusters, and each cluster was further partitioned into two sub-clusters (Figure 3). Cluster I consisted of 49 accessions and one improved variety sourced from the following regions: Amhara (37 accessions), Tigray (5 accessions and one improved variety), Afar (6 accessions), and Oromia (1 accession). Cluster II contained 50 diverse genotypes, of which 28 accessions were from Amhara, while 13 accessions, one landrace and 3 improved varieties from Tigray, 2 accessions (from Afar), 2 accessions (Oromia), and 1 accession (Gambela). Likewise, as observed from the population structure analysis, all the two main clusters and their respective sub-branches had genotypes with the potential for optimum grain yield and high oil content.

## 4. Discussion

### 4.1. Genotypic Variation and Mean Performance for Seed and Oil Yields, and Yield-Component Traits

Assessment of genetic diversity among crop genetic resources is essential to identify candidate accessions possessing desirable traits, including yield and quality attributes. The current study evaluated the genetic variation present among 100 accessions of sesame through rigorous field phenotyping and polymorphic SSR markers as a preliminary step to select genetically complementary parental accessions for breeding. 

The test genotypes showed significant (*p* ≤ 0.05) variation for grain yield and yield components (Table 3). This suggests that the germplasm pool contains vital phenotypic traits for sesame improvement through hybridization and selections. The test genotypes were sourced from five historically sesame-growing regions in Ethiopia. Given the long agricultural history and sesame production of the collection areas, it is expected that the test genotypes have adapted and evolved under local conditions through natural selection. This caused genetic differentiation of the studied sesame accessions for grain and oil yields and important yield-contributing agronomic traits. For example, the present study identified and selected sesame genotypes such as Hirhir Kebabo Hairless-9 and Setit-3 with high grain yields of >0.8 tons ha^−1^ and higher oil yields of 0.40 ton ha^−1^. The selected genotypes, which are locally referred to as Humera types, are known for their unique quality associated with product aroma and taste [30]. The selected genotypes expressed higher grain yield which is above the mean yield of 0.68 tons ha^−1^ currently recorded in Ethiopia using traditional varieties. 

### 4.2. Traits Associations

Sesame seed and oil yields are low in Ethiopia due to a lack of high-yielding varieties. These results in low financial returns for producers and processors across the sesame value chains. To improve selection response and genetic gains for economic traits, selection of highly heritable yield-contributing traits associated with seed and oil yields may be targeted in sesame improvement programs. The strong and positive correlation between seed and oil yield among the studied sesame genotypes implied both traits could be improved simultaneously in the present population. Weak correlations observed between grain yield with yield-related traits, including internode length, number of secondary branches, number of capsules per plant, stem height from base to first branch, and thousand seed weight would provide a low selection response for grain yield.

Similarly, oil yield exhibited low correlations with internode length, the number of secondary branches per plant, and thousand-seed weight implying reduced selection response for grain yield via these traits. Oil content showed poor associations with agro-morphological traits hindering direct selection. Despite the low and poor associations between seed and oil yields and oil content with yield-related agronomic traits, the present study revealed wide phenotypic variation among the studied sesame populations for several traits. These are valuable traits for future sesame phenotypic analysis, selection, and improvement in Ethiopia. Moreover, the assessed germplasm was diverse for seed and oil yields and oil content. This aided identification and selection of sesame genotypes such as Hirhir Kebabo Hairless-9, Setit-3, Orofalc, Hirhir Humera Sel-6, and Setit-1 with high seed and oil yields as useful germplasm to design and develop improved cultivars. Furthermore, sesame genotypes with relatively higher oil content, including Hirhir Humera Sel-6, Setit-1, ACC 205–180, and Orofalc ACC-2 are suitable candidates for developing new breeding populations with higher oil yield and content.

The traits accounting for the significant variation observed in the first two PCs will be important for selection. Nevertheless, 53.4% of the total variation was not explained by the PCA, probably due to the limited number of test locations used in the study. Hence, there is a need to assess the test accessions across multiple test environments and using effective molecular markers to complement the phenotypic data.

### 4.3. Genetic Diversity and Population Structure of Sesame Germplasm Based on SSR Markers

SSR markers are amongst the useful genomic resources to complement phenotypic data for effective selection. The present study recorded a mean major alleles frequency per locus of 0.78 among the sesame population (Table 6), which was much higher than values of 0.41 and 0.17 reported by [21,31] using 23 and 21 SSR among 129 Korean and 25 Ghanaian sesame genotypes, respectively. Variation in alleles frequency is attributable to genotypic differences and the number of SSR markers used in the genetic analysis [32,33,34]. The mean observed heterozygosity of 0.43 reported in the present study is lower than the value of 0.56 reported by [31] when assessing 25 sesame genotypes using 21 SSR markers. This study’s observed heterozygosity was higher than values of 0.23, 0.01, and 0.12 reported by [19,21,22] when assessing 50, 129, and 36 sesame genotypes using 10, 23, and 10 SSR markers, respectively. The mean expected heterozygosity (He = 0.30) recorded in the present study (Table 7) was lower than values of 0.72 and 0.34 reported by [21,22] when evaluating 129 and 36 sesame accessions using 23 and 10 SSR markers, respectively. The higher heterozygosity recorded in the present study suggested that the Ethiopian sesame populations have a high genetic variation for selection.

The genetic variability was confirmed by population structure analysis, which revealed four distinct populations comprising genotypes collected from different regions in Ethiopia. Most released entries (Humera-1, Setit-1, and Setit-3) were grouped in sub-population 4. Wei et al. [20] and Asekova et al. [21] reported two and three populations among 94 and 129 sesame accessions sampled from China and Korea collections using 44 and 23 SSR markers. The higher gene fixation index of 0.39 in population I comprising accessions collected from Amhara, Tigray, Afar, and Oromia regions suggest higher genetic differentiation attributable to high gene flow among these regions. Conversely, the low gene fixation index observed in population III, which comprises accessions sourced from the Amhara and Tigray regions, indicated low differentiation. This may be due to gene flow through germplasm exchange between sources of collections. The exchange of planting material regardless of geographical distances might be attributed to a low degree of differentiation in sesame populations observed in the current study.

Cluster analysis identified two major clusters and four sub-clusters, revealing genetic variation among the assessed sesame entries (Figure 3). Asekova et al. [21] grouped 129 sesame genotypes into two clusters using 23 SSR markers. In the present study, the genotypes’ clustering patterns did not correspond to the predefined population structure based on the collection regions. This may be because genotypes gathered from similar regions belong to the same gene pool or may have similar ancestral relationships [35]. Conversely, William et al. [36] reported that genetic dissimilarity among test genotypes could arise due to the diverse ancestral origin, high gene flow caused by cross-pollination and possible gene or chromosomal mutation. In this study, some sesame genotypes collected from different regions were grouped in the same cluster, such as Hirhir Kebabo Hairless Sel-6 (Tigray) and Gojam Azene (Yohans Sel-1) (Amhara), and ACC-NS-007(2) (Oromia) and GA-002(3) (Gambela) which were found in cluster I and II. In agreement with the current study, Zhang et al. [37] reported that geographical separation did not affect genetic distance among 24 sesame genotypes. Ganesamurthy et al. [38] reported that geographical separation does not affect the genetic differentiation of germplasm. Therefore, a key indicator of genetic diversity is not necessarily the geographical origin of germplasm collections. The exchange of genetic materials among farmers and traders in the regions contributes to high gene flow and a lack of genetic differentiation. Barnaud et al. [39], suggested that farmers’ selections and management practices affect genetic diversity patterns.

To develop new breeding populations possessing desirable agronomic traits, especially high grain and oil yields, crosses could be made between distantly related and complementary genotypes selected from different clusters. For instance, for improved grain and oil yields, the following entries were selected such as Setit-3, Orofalc ACC-2, Hirhir Humera Sel-6, ACC-NS-007(2), Hirhir Kebabo Hairless-9, and ACC 205-180. These genotypes are localized in sub-cluster II-a and sub-cluster II-b. The two clusters contained candidates with excellent grain and oil yields.

In conclusion, the current study determined the extent of genetic variation among 100 diverse sesame germplasm collections of Ethiopia using phenotypic traits and simple sequence repeat (SSR) markers to select distinct and complementary parents for breeding. The test genotypes exhibited significant phenotypic variation for key agronomic traits including grain yield, oil content, and oil yield, which were underpinned by their genetic diversity. The sesame genotypes were differentiated into four major populations based on the model-based population structure analysis. The moderate heterozygosity and fixation index among the accessions suggests that the accessions have distinct heterotic groups desirable for breeding. Based on wide genetic divergence, the following genotypes were selected for use in future sesame breeding programs: Hirhir Humera Sel-6, Setit-3, Hirhir Kebabo Hairless Sel-4, Hirhir Nigara 1st Sel-1, Humera-1, Orofalc ACC-2, and Hirhir Kebabo Early Sel-1 (selected from subgroup II-a), Hirhir kebabo hairless-9, NN-0029(2), NN0068-2, Hirhir Filwha Large Seeded, and Bawnji Fiyel Kolet, (from subgroup II-b). Progeny development and field evaluation by combining ability analysis are recommended among the selected parents to establish heterotic groups for sesame pre-breeding.

## Figures and Tables

**Table 1:** Names and sources of 100 sesame accessions included the study.

Entry Number	Entry Name or Designation	Description	Source (Regions or Research Center in Ethiopia)	Entry Number	Entry Name or Designation	Description	Source (Regions or Research Center in Ethiopia)
1	Hirhir Kebabo Hairless Sel-2	Accession	Tigray	51	Hirhir Baeker-Sel-3	Accession	Tigray
2	GXT = 85(28-2)	Accession	Afar	52	NN0068-3	Accession	Amhara
3	Hirhir Kebabo Hairless-9	Accession	Tigray	53	NN0074-3	Accession	Amhara
4	NN-0068-1	Accession	Amhara	54	NN0036-1	Accession	Amhara
5	NN-0108-2	Accession	Amhara	55	Hirhir Kebabo Hairless Sel-4	Accession	Tigray
6	NN-034	Accession	Amhara	56	NN0001-2	Accession	Amhara
7	BCS-0041	Accession	Afar	57	Bawnji Sel-2	Accession	Amhara
8	ACC-031-5-14	Accession	Tigray	58	NN0058-2	Accession	Amhara
9	NN-0129-2	Accession	Amhara	59	G-02	Accession	Amhara
10	ACC-203-020	Accession	Amhara	60	ACC-NS-007(2)	Accession	Oromia
11	NN-0038-2	Accession	Amhara	61	GA-002(3)	Accession	Gambela
12	Bawnji Fiyel Kolet	Accession	Amhara	62	Endelemi Kirem Sel-2	Accession	Amhara
13	Gojam Azene (Aleka)	Accession	Amhara	63	ACC-205-299	Accession	Tigray
14	Hirhir Humera Sel-6	Accession	Tigray	64	Hirhir Kebabo Early Sel-1	Accession	Tigray
15	Bawnji Gobate	Accession	Amhara	65	NN0016-1	Accession	Amhara
16	Shwarobit (83)	Accession	Amhara	66	Hirhir Adgeshu Sel -8	Accession	Tigray
17	Humera-1	Variety	HuARC ^†^	67	NN0015	Accession	Amhara
18	ACC-202-950	Accession	Amhara	68	NN01-13	Accession	Amhara
19	NN-0026	Accession	Amhara	69	Bering Bawany	Accession	Afar
20	ACC-NO-041	Accession	Tigray	70	Hirhir Nigara 1st Sel-2	Accession	Tigray
21	ACC-203-612	Accession	Amhara	71	Gojam Azene (Yohans Sel-1)	Accession	Amhara
22	ACC-200-064-1	Accession	Tigray	72	NN0038-1	Accession	Amhara
23	Setit-1	Variety	HuARC	73	NN0104	Accession	Amhara
24	Tejareb Kokit Sel-3	Accession	Amhara	74	Hirhir Kebabo Hairless Sel-6	Accession	Tigray
25	Orofalc ACC-2	Accession	Afar	75	ACC 205-180	Accession	Tigray
26	NN-0022	Accession	Amhara	76	Hirhir	Landrace	Tigray
27	Setit-3	Variety	HuARC	77	ACC 203-616	Accession	Amhara
28	ABX = 2-01-2	Accession	Afar	78	NN0025	Accession	Amhara
29	NN-0020	Accession	Amhara	79	NN-0183-3	Accession	Amhara
30	ACC-NS-010	Accession	Oromia	80	Hirhir Humera	Accession	Tigray
31	Hirhir Sel-2	Accession	Tigray	81	NN0031	Accession	Amhara
32	ABXT-85-SEL-2-1	Accession	Afar	82	NN0061	Accession	Amhara
33	Setit-2	Variety	HuARC	83	ABXC-50402	Accession	Afar
34	Hirhir Kebabo Hairless-Sel-7	Accession	Tigray	84	NN0021	Accession	Amhara
35	NN-0143	Accession	Amhara	85	NN0079-1	Accession	Amhara
36	ACC NS-031	Accession	Oromia	86	ACC 202-333	Accession	Amhara
37	NN-0029(2)	Accession	Amhara	87	NN-0052	Accession	Amhara
38	NN-0054	Accession	Amhara	88	NN-0029-1	Accession	Amhara
39	Morgo-Sel-P = 13	Accession	Afar	89	Teiahir Sanja Sel-6	Accession	Amhara
40	Hirhir Humera Sel-8	Accession	Tigray	90	ACC-202-358	Accession	Amhara
41	Tejareb Girar	Accession	Amhara	91	NN0032	Accession	Amhara
42	NN0027	Accession	Amhara	92	NN0071	Accession	Amhara
43	NN0009	Accession	Amhara	93	NN0064-1	Accession	Amhara
44	ACC-203-610	Accession	Amhara	94	NN0056	Accession	Amhara
45	NN-0146	Accession	Amhara	95	NN-01-03	Accession	Amhara
46	NN-0044-2	Accession	Amhara	96	NN0032-2	Accession	Amhara
47	NN-0018-2	Accession	Amhara	97	Bawnji Maksegnt	Accession	Amhara
48	Hirhir Nigara 1st Sel-1	Accession	Tigray	98	Bawnji Flwha Sel-2	Accession	Amhara
49	NN00136-1	Accession	Amhara	99	NN0068-2	Accession	Amhara
50	NN-0088-2	Accession	Amhara	100	Hirhir Filwha Large Seeded	Accession	Amhara

^†^ HuARC, Humera Agricultural Research Centre. Tigray, Amhara, Afar, Oromia, and Gambela are administrative regions in Ethiopia.

**Table 2:** Description of the SSR markers used in the study.

No	Primers	Forward Primer Sequence	Reverse Primer Sequence	Product Size (bp)
1	ID0046	TCAACGTGATTGCTCCCATA	CAGCTGCCTGAAAGAAGAGG	101
2	ZMM1043	CCCGAAAATAGGATTTCTAACCA	TTTTGGACTGCTATTGAGGGA	184
3	ZMM3261	CGAAAGCATGAGACGAGTATG	AACTAGTGCGCAATTCATTCAA	244
4	ID0041	AGGCTTTCACATCATCAAATG	CATGTAGGATGCAACTCTTCAAA	280
5	ZMM5015	ATTTATTGGGTTGCTGGGAA	TGAAAATTAAGTCACCAGTACCACC	151
6	ZMM4664	CCTTCACTTCAAATCCGTCAA	TTTGGTTTGCATAGATGCTCTT	184
7	ZMM1809	TTAAGCCTCGTTGACTCCAA	ATTGTACGGCATGTTGTCCC	256
8	ZMM2321	CAACACCACCAACGCATATC	AGCAACGATTCACGACATTG	280
9	ZMM5358	TAGGATGCTTTGAATTGGGC	AGGAACAAACATACGGCGTC	164
10	ID0068	TCTTCGGAGTTAACACCCTCA	TTGGATTTCCATGTATGCCA	199
11	ZMM3312	GCAAAATCTTCTTTCCTCCG	GCAGCAAGGGAATTGAATGT	264
12	ZMM1033	CGTAGTGGTTCCCCTCACAT	ATGCTTTCCCCCAAATAACC	179
13	ZMM1189	TATCCAGGGGAAAACCAGAA	TTGGATTTTCCTTCTCACGC	212
14	ZMM2202	TCAGGAAGAAAGAATTGCTGC	CAATTTAACCATCCTGACTC	276
15	ZMM1637	GCGGTGACATATTAAGGGCA	ACCGGAATCCGAACATGTAA	265
16	ZMM4645	TTGAGCGATTCATCGACTTG	TTCTCCGGCCATTTTAATCA	179
17	ZMM1700	CATTAACACCATTACGCAAACA	TTTGGCAAAACTAGCAATGAA	258
18	ID0175	CAATTTTGATTTCTTTATCTATTTTCG	TCGAGTGCCCGAATTTTAAG	271
19	ZMM1353	GCCAAAACAAAGGATTCAAGA	TGAGCTTTGTGTGACCATGA	169
20	ID0145	ACCCTCCCTCCATGAATTTT	CCTCCATCTCATCTCATCCC	196
21	ZMM4803	TGCATGAGCTAAGGGAAAGG	TGGTGGCAATTTGCAAGTAA	268
22	ZMM6141	AAAAAGCAAAATCCATAATTTGA	TTGCCCCCTCAACTATTTG	167
23	ZMM3013	TGCCAGTTGGCATATACCATTA	GAGCCGGTCTGAAATTTATCC	216
24	ZMM2818	CGTGTGCCCAATATTTGAGTT	TCAACCTCCTCCCTACACAA	279
25	ZMM3223	CGATGGTTATTAAATTAAGTATTCGG	GACATTTGAAGCAAAGTGTATCG	279
26	ZMM1691	CTTGACCTGGAGTGTACGGC	GGATCAAACAGACACGAGCA	220
27	ZMM1851	TGACTCTTTCGATTTGGGCT	CGAAAAATACGGGCGTTACT	280

Source: Wei et al. [20], bp = base pairs.

**Table 3:** Analysis of variance showing mean square values and level of significance for the studied agro-morphological characters, and oil yield of 100 sesame collections evaluated in two locations in Ethiopia.

Traits														
Source of Variation	d.f.	DF	DM	PH	INL	NPB	NSB	NCPP	NSPC	SHB	DFLBC	TSW	GYH	OYH
Rep (Loc)	2	171.27	205.65	714.76	0.00	121.72	28.62	14,855.85	522.58	1730.39	374.90	17.61	0.00	0.06
Block (Loc*Rep)	36	5.05	16.04	240.28	1.16	1.15	0.14	276.21	249.16	70.61	111.98	6.36	0.05	0.07
Entry (Gen)	99	6.93 *	16.46 *	360.33 *	2.87 **	0.61 ns	1.24 **	145.00 ns	189.08 **	70.52 ns	122.26 **	5.43 ns	0.08 **	0.07 ns
Location (Env)	1	201.64 **	60.06 *	423.33 ns	852.35 **	39.06 **	35.40**	116.64 ns	2787.84 **	11,306.07 **	36,898.56 **	1.32 ns	3.94 **	1.47 **
Gen × Env	99	5.05 ns	14.03 ns	336.30 *	3.43 **	0.67 *	0.68 *	179.46 ns	136.61 ns	48.88 ns	107.58 **	5.49 ns	0.70 **	0.05 ns
Error	162	4.69	11.08	243.30	1.54	0.47	0.40	143.65	112.01	52.94	65.88	5.37	0.03	0.05

Note: Genotype by environment interaction (Gen × Env), * and ** denote significance difference at the 5% and 1% levels of probability, respectively; Non-significant (NS), Degrees of freedom (DF), Days-to-50% flowering (DF), Days-to-75% maturity (DM), Plant height (PH) (cm), Inter node length (INL) (cm), Number of primary branches per plant (NPB), Number of secondary branches per plant (NSB), Number of capsules per plant (NCPP), Number of seeds per capsule (NSPC), Stem height to 1st branch (SHB) (cm), Distance from lowest branch to 1st capsule (DFLBC)(cm), Thousand-seed weight (TSW) (g/1000 seed), Grain yield per hectare (GYH) (ton ha^−1^), Oil yield per hectare (OYH) (ton ha^−1^).

**Table 4:** Mean values for agronomic traits of 100 sesame genotypes of Ethiopia showing the top 10 and bottom 5 ranked entries based on grain yield (ton ha^−1^) across two sites.

Traits															
No	Entry Name or Designation	DF	DM	PH	INL	NPB	NSB	NCPP	NSPC	SHB	DFLBC	TSW	GYH	OYH	OC
	*Top 10 entries*														
1	Hirhir Kebabo Hairless-9	41 ^bcd^	90 ^cd^	123.9 ^abc^	9.8 ^abc^	4 ^a^	1 ^d^	41 ^abcd^	58 ^abcd^	18.1 ^cd^	42.8 ^ab^	3.0 ^a^	1.01 ^a^	0.40 ^a^	50.9
2	Setit-3	43 ^abc^	91 ^bcd^	131.6 ^a^	10.0 ^ab^	3 ^b^	2^c^	52 ^a^	63 ^a^	18.0 ^cd^	26.5 ^cd^	3.1 ^a^	0.84 ^ab^	0.40 ^a^	49.1
3	Orofalc ACC-2	42 ^abcd^	89 ^d^	103.9 ^cd^	8.3 ^bcd^	3 ^b^	2 ^c^	36 ^abcd^	56 ^abcd^	13.8 ^d^	22.4 ^d^	3.1 ^a^	0.80 ^ab^	0.40 ^a^	52.5
4	Hirhir Humera Sel-6	43 ^abc^	92 ^bcd^	114.8 ^abcd^	9.7 ^abc^	4 ^a^	4 ^a^	48 ^abc^	61 ^abc^	27.2 ^abc^	38.1 ^ab^	2.9 ^a^	0.78 ^ab^	0.39 ^ab^	53.9
5	ABX = 2-01-2	44 ^ab^	95 ^ab^	133.2 ^a^	8.7 ^bcd^	4 ^a^	3 ^b^	40 ^abcd^	65 ^a^	18.9 ^bcd^	40.9 ^ab^	2.7 ^a^	0.74 ^b^	0.36 ^abc^	48.9
6	Setit-1	40 ^cd^	88 ^d^	125.6 ^ab^	7.9 ^d^	4 ^a^	2 ^c^	39 ^abcd^	56 ^abcd^	21.9 ^bcd^	38.9 ^ab^	3.3 ^a^	0.73 ^b^	0.39 ^ab^	53.8
7	ACC 205-180	45 ^a^	97 ^a^	126.9 ^ab^	10.5 ^a^	3 ^b^	2 ^c^	40 ^abcd^	55 ^abcd^	32.5 ^a^	42.0 ^ab^	3.1 ^a^	0.72 ^b^	0.38 ^ab^	53.1
8	ACC 203-616	45 ^a^	94 ^abc^	106.8 ^bcd^	8.5 ^bcd^	3 ^b^	1 ^d^	37 ^abcd^	54 ^abcd^	25.5 ^abc^	39.0 ^ab^	2.6 ^a^	0.69 ^b^	0.35 ^abc^	51.7
9	NN-0029(2)	40 ^cd^	88 ^d^	118.5 ^abcd^	9.5 ^abcd^	4 ^a^	1 ^d^	44 ^abcd^	65 ^a^	28.2 ^abc^	41.9 ^ab^	3.4 ^a^	0.68 ^b^	0.36 ^abc^	50.7
10	GA-002(3)	42 ^abcd^	90 ^cd^	119 ^abcd^	10.3 ^ab^	4 ^a^	2 ^c^	49 ^ab^	63 ^a^	27.7 ^abc^	34.8 ^bc^	2.8 ^a^	0.67 ^b^	0.33 ^abc^	49.3
	*Bottom 5 entries*														
1	NN-0183-3	43 ^abc^	92 ^bcd^	117.1 ^abcd^	8.1 ^cd^	3 ^b^	2 ^c^	34 ^bcd^	48 ^bcd^	20.8 ^bcd^	42.6 ^ab^	2.3 ^a^	0.17 ^c^	0.07 ^bc^	45.8
2	NN-0020	43 ^abc^	98 ^a^	125.8 ^ab^	8.2 ^cd^	4 ^a^	2 ^c^	34 ^bcd^	62 ^ab^	29.0 ^ab^	44.4 ^ab^	2.6 ^a^	0.24 ^c^	0.12 ^abc^	49.3
3	NN-0108-2	39 ^d^	90 ^cd^	135.2 ^a^	10.2 ^ab^	4 ^a^	0 ^e^	32 ^cd^	47 ^cd^	20.6^bcd^	47.2 ^a^	2.9 ^a^	0.26 ^c^	0.04 ^c^	47.3
4	NN00136-1	43 ^abc^	97 ^a^	99.6 ^d^	10.2 ^ab^	3 ^b^	2 ^c^	42 ^abcd^	44 ^d^	26.1 ^abc^	47.4 ^a^	3.0 ^a^	0.26 ^c^	0.13 ^abc^	47.5
5	NN-0143	42 ^abcd^	94 ^abc^	132.2 ^a^	10.3 ^ab^	4 ^a^	3 ^b^	30 ^d^	55 ^abcd^	27.2 ^abc^	39.7 ^ab^	3.2 ^a^	0.28 ^c^	0.15 ^abc^	48.3
	Mean	43	92	119.6	9.2	3.5	2	41	51	24.4	39.8	2.9	0.48	0.24	49.7
	CV (%)	5.03	3.61	13.30	13.52	19.21	38.58	29.42	20.92	29.74	20.44	78.06	35.26	90.13	NA
	R^2^ (%)	72.87	70.33	68.59	86.27	85.16	82.97	77.76	70.65	77.22	85.95	60.98	82.15	66.42	NA

Note: Coefficient of variation (CV), Coefficient of determination (R^2^), Not available (NA), Days-to-50% flowering (DF), Days-to-75% maturity (DM), Plant height (PH) (cm), Inter node length (INL) (cm), Number of primary branches per plant (NPB), Number of secondary branches per plant (NSB), Number of capsules per plant (NCPP), Number of seeds per capsule (NSPC), Stem height to 1st branch (SHB) (cm), Distance from lowest branch to 1st capsule (DFLBC)(cm), Thousand-seed weight (TSW) (g/1000 seed), Grain yield per hectare (GYH) (ton ha^−1^), Oil yield per hectare (OYH) (ton ha^−1^), Oil content (OC) (%), Means in a column followed by the same letter are not significantly different at the 5% probability level of Tukey’s Honestly Significant Difference.

**Table 5:** Phenotypic correlations coefficients for assessed agro-morphological traits, oil content, and oil yield of 100 sesame collections evaluated across two locations in Ethiopia.

Traits	DM	PH	INL	NPB	NSB	NCPP	NSPC	SHB	DFLBC	TSW	OC	OYH	GYH
DF	0.31 **	0.05 ns	0.14 **	0.09 ns	0.09 ns	0.16 **	−0.21 **	0.05 ns	−0.13 **	−0.02 ns	−0.11 *	0.08 ns	0.09 ns
DM	1.00	0.07 ns	0.08 ns	−0.20 ns	0.07 ns	−0.10 *	−0.12 *	0.09 ns	0.00 ns	−0.15 **	−0.06 ns	−0.10 *	−0.09 ns
PH		1.00	0.09 ns	0.06 ns	0.12 *	0.07 ns	0.05 ns	0.01 ns	0.07 ns	0.10 *	−0.00 ns	0.05 ns	0.05 ns
INL			1.00	−0.17 **	0.21 **	0.08 ns	−0.08 ns	0.38 **	−0.45 **	0.00 ns	0.03 ns	0.35 **	0.35 **
NPB				1.00	0.06 ns	0.30 **	0.08 ns	−0.14 **	0.25 **	0.20 **	0.05 ns	−0.05 ns	−0.06 ns
NSB					1.00	−0.05 ns	0.04 ns	0.34 **	−0.166 **	−0.122 *	0.051 ns	0.214 **	0.207 **
NCPP						1.00	0.119 *	−0.00 ns	−0.01 ns	0.16 **	−0.01 ns	0.17 **	0.18 **
NSPC							1.00	−0.07 ns	0.21 **	0.04 ns	−0.08 ns	0.15 **	0.17 **
SHB								1.00	−0.26 **	−0.13 **	0.02 ns	0.16 **	0.16 **
DFLBC									1.00	−0.01 ns	−0.02 ns	−0.29 **	−0.29 **
TSW										1.00	0.05 ns	0.23 **	0.23 **
OC											1.00	0.16 **	0.06 ns
OYH												1.00	0.99 **

Note: * and ** denote significant difference at the 5% and 1% levels of probability, respectively; Non-significant (NS), Days-to-50% flowering (DF), Days-to-75% maturity (DM), Plant height (PH) (cm), Inter node length (INL) (cm), Number of primary branches per plant (NPB), Number of secondary branches per plant (NSB), Number of capsules per plant (NCPP), Number of seeds per capsule (NSPC), Stem height to 1st branch (SHB) (cm), Distance from lowest branch to 1st capsule (DFLBC)(cm), Thousand-seed weight (TSW) (g/1000 seed), Oil content (OC) (%), Oil yield per hectare (OYH) (ton ha^−1^), Grain yield per hectare (GYH) (ton ha^−1^).

**Table 6:** Genetic parameters estimated for 100 sesame genotypes using 27 SSR markers.

Locus	Product Size (bp)	Genetic Parameter			
		MAF	He	Ho	PIC
ID0046	101	0.72	0.40	0.56	0.32
ZMM1043	184	0.75	0.38	0.51	0.31
ZMM3261	244	0.59	0.48	0.82	0.37
ID0041	280	0.96	0.08	0.08	0.07
ZMM5015	151	0.79	0.34	0.43	0.28
ZMM4664	184	0.60	0.48	0.80	0.36
ZMM1809	256	0.86	0.24	0.28	0.21
ZMM2321	280	0.90	0.18	0.20	0.16
ZMM5358	164	0.62	0.47	0.77	0.36
ID0068	199	0.86	0.25	0.29	0.22
ZMM3312	264	0.56	0.49	0.89	0.37
ZMM1033	179	0.76	0.37	0.49	0.30
ZMM1189	212	0.52	0.50	0.96	0.37
ZMM2202	276	0.89	0.20	0.22	0.18
ZMM1637	265	0.68	0.44	0.65	0.34
ZMM4645	179	0.81	0.31	0.39	0.26
ZMM1700	258	0.95	0.10	0.10	0.09
ID0175	271	0.96	0.08	0.08	0.07
ZMM1353	169	0.94	0.12	0.13	0.11
ID0145	196	0.77	0.36	0.47	0.29
ZMM4803	268	0.95	0.10	0.11	0.10
ZMM6141	167	0.75	0.38	0.51	0.31
ZMM3013	216	0.69	0.43	0.63	0.34
ZMM2818	279	0.96	0.08	0.08	0.07
ZMM3223	279	0.82	0.30	0.36	0.25
ZMM1691	220	0.73	0.39	0.54	0.32
ZMM1851	280	0.90	0.18	0.20	0.16
Mean	221	0.78	0.30	0.43	0.25

MAF = Major allele frequency, He = Unbiased expected heterozygosity (gene diversity), Ho = Observed heterozygosity, PIC = Polymorphic information content.

**Table 7:** Genetic clusters and their member entries, the proportion of the membership, mean expected heterozygosity, and fixation index based on structure analysis of 63 sesame entries with 27 SSR markers.

Population	Entries	Membership %	Expected Heterozygosity	Mean Fixation Index
I	ACC NS-031E63, Hirhir Nigara 1st Sel-2, Hirhir Kebabo Hairless Sel-6, NN-0183-3, GXT = 85(28 − 2), ABXT-85-Sel-2-1, ABXC-50402, NN-0129-2, ACC-203-020, Gonjam Azene (Aleka), NN-0026, Tejareb Kokit Sel-3, NN0027, NN0009, NN-0088-2 Bawnji Sel-2, G-02, Endelemi kirem sel-2, NN0016-1, NN0038-1, NN-0052, NN0071, NN0064-1	24	0.15	0.39
II	Hirhir Baeker-Sel-3, NN0025, BCS-0041, Orofalc ACC-2, ABX = 2-01-2, Bering Bawany, ACC-203-612, NN-0143, NN-0146, NN-0044-2, NN-0018-2, NN-0029-1, NN0056	13	0.22	0.23
III	Hirhir kebabo hairless sel-2, Hirhir kebabo hairless-9, ACC-200-064-1, HIRHIR NIGARA 1ST SEL-1, Bawnji Fiyel Kolet, NN0104, Bawnji Maksegnt, Bawnji Flwha Sel-2, Hirhir Filwha Large Seeded	9	0.29	0.02
IV	Hirhir Humera Sel-6, Humera-1, Setit-1, Setit-3, Hirhir Sel-2, Hirhir Kebabo Hairless-Sel-7, Hirhir Humera Sel-8, NN0036-1, Hirhir Kebabo Early Sel-1, Hirhir Adgeshu Sel -8, Morgo-Sel-P = 13, NN-0020 ACC-203-610, NN0001-2, NN01-13, Gojam Azene(Yohans Sel-1), NN0031	17	0.20	0.29

## Data Availability

All data generated or analysed during this study are included in this published article.
